# Intercostal Nerve Block in Uniportal Video-Assisted Thoracoscopic Surgery: A Propensity-Score Matched Single-Center Study of Early Postoperative Pain and Opioid Use

**DOI:** 10.3390/jcm15134910

**Published:** 2026-06-24

**Authors:** Fahim Kanani, Narmin Zoabi, Eduard Khabarov, Zoey Berdan, Moshe Argaman, Mirit Meller, Rijini Nugzar, Oren Fruchter, Mohammad Eid Al Mohtasib, Mordechai Shimonov, Anas Salhab, Moshe Kamar, Firas Abu Akar

**Affiliations:** 1 Department of Surgery, Edith Wolfson Medical Center, Holon 58100, Israel; kanani.fahim@gmail.com (F.K.); dreduardkhabarov@gmail.com (E.K.); zoeybardan98@gmail.com (Z.B.); moshearg@gmail.com (M.A.); miritmel8@gmail.com (M.M.); dr.kamar.moshe@gmail.com (M.K.); 2 Sackler Faculty of Medicine, Tel Aviv University, Tel Aviv 6997801, Israel; zoabinarmin@gmail.com (N.Z.); prof.fruchter@gmail.com (O.F.);; 3 Department of Gastroenterology Shiba Medical Center, Ramat Gan 5265601, Israel; 4 Department of Thoracic Surgery, The Edith Wolfson Medical Center, 62 Ha-Lokhamim Street, Holon 58100, Israel; 5 Department of Anaesthesia, The Edith Wolfson Medical Center, Holon 58100, Israel; 6 Department of Pneumonology, The Edith Wolfson Medical Center, Holon 58100, Israel

**Keywords:** intercostal nerve block, uniportal VATS, postoperative pain, opioid-sparing, propensity-score matching

## Abstract

**Background**: Acute pain after video-assisted thoracoscopic surgery (VATS) promotes respiratory splinting, impaired cough, and pulmonary complications, and predicts persistent opioid use. Surgeon-administered intercostal nerve block (ICNB) is a simple regional technique, but its independent effect on early pain and opioid requirement in a contemporary uniportal VATS (UVATS) pathway is incompletely defined. **Methods**: We performed a retrospective cohort study of 456 consecutive patients undergoing UVATS at a single Israeli center between 2017 and 30 May 2025. Patients receiving an intercostal block were compared with those who did not. Baseline covariates were balanced by 1:1 nearest-neighbor propensity-score matching (caliper 0.2 SD of the logit propensity score). The primary endpoints were pain on postoperative day (POD) 1 (visual analog scale, VAS) and postoperative opioid use; secondary endpoints included later pain, analgesic regimen, postoperative pneumonia, and mortality. **Results**: Matching yielded 159 patients per group (*n* = 318) with all clinically relevant covariates balanced (standardized mean difference [SMD] < 0.13). Median POD1 VAS was lower with the block (4 [IQR 3–4] vs. 5 [5–7]; *p* < 0.001), and 76.1% of block patients were opioid-free versus 10.7% who were not (*p* < 0.001). The effect was concentrated early and attenuated by POD3. In multivariable analysis the block was independently associated with lower POD1 VAS (adjusted β = −1.64, 95% CI −2.00 to −1.29; *p* < 0.001). Postoperative pneumonia was less frequent in the block group (5.7% vs. 20.1%; *p* < 0.001). Thirty-day and one-year mortality did not differ significantly. **Conclusions**: In UVATS, a surgeon-placed intercostal nerve block was associated with lower early postoperative pain that persisted after adjustment for operating surgeon and surgical era, concordant with pooled meta-analytic estimates; associated reductions in opioid use and pneumonia were confounded with surgeon and secular trend and are hypothesis-generating. These single-center, retrospective findings require prospective, protocol-randomized confirmation.

## 1. Introduction

Although video-assisted thoracoscopic surgery (VATS) is minimally invasive, patients still experience moderate-to-severe acute postoperative pain arising from skin incision, intercostal muscle and nerve trauma, rib retraction, and chest-drain irritation [1]. Inadequate control of this pain initiates a well-characterized cascade: nociception promotes respiratory splinting and an ineffective cough, which in turn predispose to atelectasis and pneumonia, limit early mobilization, prolong hospital stay, and increase the likelihood of chronic post-thoracotomy pain and persistent opioid use [1,2]. Regional analgesia interrupts this cascade by blocking somatic nociceptive input from the chest wall and pleura, reducing systemic opioid requirements and the attendant risks of respiratory depression, nausea, and ileus while preserving pulmonary mechanics [1].

Among regional options, the intercostal nerve block (ICNB) occupies a distinctive position. Compared with thoracic epidural analgesia (TEA) and paravertebral block (PVB), it offers marked procedural simplicity: it can be performed rapidly by the surgeon under direct thoracoscopic vision at the conclusion of the operation, without ultrasound guidance and with a negligible learning curve [3]. It also carries a favorable safety profile, avoiding the neuraxial hazards of TEA and the sympathectomy-mediated hypotension and urinary retention associated with central techniques [3]. Relative to systemic opioids alone, ICNB consistently improves early analgesia and reduces opioid consumption [4].

The literature is nonetheless mixed on where ICNB ranks among regional techniques. There is broad agreement that ICNB is superior to systemic analgesia and is associated with fewer side effects and lower failure rates than TEA [1,4]. Its standing relative to other blocks, however, is contested: a recent randomized trial found ICNB noninferior to TEA for pain [5,6], whereas several trials and reviews rank PVB as superior to ICNB for 24 h pain and opioid consumption [7,8], and the PROSPECT procedure-specific guideline does not recommend ICNB as a first-line technique, citing insufficient procedure-specific evidence and favoring PVB or erector spinae plane block [2]. Newer ultrasound-guided fascial-plane techniques—the erector spinae plane and serratus anterior plane blocks—have gained popularity as alternatives, with the serratus anterior plane block reported to provide longer-lasting analgesia at 24–48 h [3,9]. A central, recurring limitation of single-shot ICNB is its short duration of action—approximately 6–8 h owing to rapid systemic absorption from the vascular intercostal space—which leaves patients vulnerable to rebound pain by 24–48 h unless long-acting formulations are used [4,10].

The relevant question in contemporary practice is therefore not whether regional analgesia reduces pain, but what marginal benefit a surgeon-placed intercostal block adds within a uniportal pathway that is already minimally invasive. Reducing the operation to a single incision does not eliminate chest-wall nociception; it concentrates instrument-related compression and leverage on one intercostal level, providing a clear mechanistic rationale for a level-targeted block even when only one incision is made. Real-world data on ICNB specifically within a contemporary uniportal VATS (UVATS) pathway nonetheless remain limited, much of the comparative evidence deriving from heterogeneous multi-institutional populations differing in surgical approach, anesthetic agent, and block timing [5]. We therefore report a single-center observational cohort describing early postoperative pain, opioid use, and pulmonary outcomes in UVATS patients managed with and without a standardized surgeon-administered intercostal block. As detailed in the Methods, block use at our center was strongly patterned by operating surgeon and surgical era; we accordingly examined and adjusted for this confounding, and frame the early analgesic effect as the primary finding while presenting the opioid-sparing and pulmonary signals as hypothesis-generating. The study question is summarized in PECO format (Table 1).

## 2. Methods

### 2.1. Study Population and Design

We conducted a retrospective cohort study of 600 consecutive adult patients who underwent uniportal VATS in a single center, between 2017 and 30th of May 2025. The study was approved by the institutional review board (WOMC-0082-25 on 28 June 2025); the requirement for individual informed consent was waived owing to the retrospective design. Patients were eligible if they underwent UVATS for any indication during the study period. The exclusion criteria were, conversion to open thoracotomy at outset, incomplete primary-outcome data, age <18, chronic pain and known infiltration of cancer to the chest wall. Patients were classified into two groups according to whether they received an intercostal nerve block: the **block** group and the **no-block** group.

### 2.2. Intercostal Nerve Block Protocol

In the block group, an intercostal nerve block was administered intraoperatively by the operating surgeon under direct thoracoscopic visualization at the conclusion of the procedure, before chest closure. A mixture of lidocaine 2% (10 mL, 200 mg) and bupivacaine 0.5% (10 mL, 50 mg) was injected under thoracoscopic vision at three intercostal levels—the intercostal space at the level of the utility incision, together with the space immediately above (the rib before) and the space immediately below (the rib after)—with a total of 20 mL of the mixture distributed across the three levels. These total doses (lidocaine 200 mg; bupivacaine 50 mg) were within the recommended maximum single doses for adults (lidocaine ≤ 4.5 mg/kg; bupivacaine ≤ 2 mg/kg) for any patient above approximately 45 kg, and no weight-based dose adjustment was required in this cohort. Injections were placed at the posterior aspect of the intercostal space, and were deep, toward the parietal pleura along the inferior border of the rib. The identical anesthetic mixture, total volume, and three-level technique were applied to all block-group patients according to a single institutional protocol. The no-block group underwent the same operation and perioperative pathway without intercostal infiltration.

### 2.3. Anesthetic and Perioperative Care

All patients underwent uniportal VATS under general anesthesia with single-lung ventilation via a double-lumen endotracheal tube or bronchial blocker, with intraoperative analgesia provided by short-acting opioids titrated by the attending anesthesiologist. A single intercostal chest drain was placed at the end of the procedure. Postoperatively, all patients received a standardized multimodal analgesic regimen comprising scheduled non-opioid analgesia (paracetamol and a nonsteroidal anti-inflammatory agent, where not contraindicated), with opioids administered as rescue analgesia for breakthrough pain; scheduled and rescue analgesics were escalated at the discretion of the treating team according to patient-reported pain. Early mobilization and chest physiotherapy were encouraged from the first postoperative day.

### 2.4. Pain Assessment

Postoperative pain was assessed by ward nursing staff using a standard 0–10 visual analog scale (VAS: 0 = no pain; 10 = worst imaginable pain) as part of routine postoperative care. Scores were recorded at rest, typically twice per nursing shift (approximately every 8 h), from the first postoperative day until discharge. Because patients were managed across multiple surgical wards, several measurements could be available within a given day; the highest recorded value was retained per day, and the highest daily value within each window was used as the patient’s score for that window. Scores were aggregated into early (POD1–2) and late (POD3 and beyond) windows. Because this was a retrospective study based on routinely collected clinical data, pain assessors were not blinded to treatment allocation.

Postoperative opioid use refers to opioids administered on the ward during the postoperative admission period only. Intraoperative agents—including intraoperative fentanyl given as part of the anesthetic—were not counted toward the postoperative opioid or opioid-free classification; the fentanyl category in Table 2 therefore denotes postoperative ward administration, distinct from intraoperative anesthetic dosing. “Opioid-free” denotes the absence of any postoperative ward opioid. Opioid exposure was recorded categorically (opioid-free status and specific agent); per-dose quantities were not uniformly available, and morphine milligram equivalents were therefore not computed.

### 2.5. Variables and Endpoints

Baseline variables comprised age, sex, body mass index (BMI), smoking status, ASA physical status, Mallampati class, underlying pulmonary disease category, malignancy, and comorbidities (hypertension, diabetes mellitus, ischemic heart disease, COPD, chronic renal failure, liver disease, rheumatic disease). Operative variables included operation group, operative time, arterial-line placement, conversion to intubation, and need for intubation.

The **primary endpoints** were (i) pain on POD1, recorded on a 0–10 visual analog scale (VAS), and (ii) postoperative opioid use, expressed as the proportion of patients managed without any postoperative ward opioid (“opioid-free”). **Secondary endpoints** were pain on POD3 and beyond, total pain duration, analgesic class and route, postoperative pneumonia, pulmonary-toilet-related events, and 30-day and one-year mortality.

Postoperative pneumonia was defined as a new or progressive pulmonary infiltrate on chest radiography or computed tomography occurring after surgery, accompanied by at least one compatible clinical feature—fever (>38 °C), leukocytosis (>12 × 10^9^/L) or leukopenia (<4 × 10^9^/L), purulent sputum, increased respiratory secretions, or a clinical decision to initiate antibiotics for suspected lower respiratory tract infection. Cases were identified through a review of clinical records, radiological reports, and discharge documentation. These criteria were applied uniformly across the study period. Radiographic confirmation (chest radiography or computed tomography) was a required component of the definition. As a retrospective study, outcome ascertainment was based on the clinical record and was not performed by assessors blinded to treatment allocation; the potential for ascertainment bias is addressed in the Discussion.

### 2.6. Statistical Analysis

Continuous variables are presented as median [interquartile range, IQR] and compared with the Mann–Whitney U test; categorical variables are presented as counts (percentages) and compared with the chi-square or Fisher exact test. Covariate balance was assessed using the standardized mean difference (SMD), with values <0.20 considered acceptable balance.

Missing baseline covariate data were handled by multiple imputations (which were performed before propensity-score estimation and matching; the treatment indicator and outcomes were not included in the imputation models) by chained equations (predictive mean matching for continuous variables, logistic and polytomous regression for categorical variables), restricted to covariates with ≤40% missingness; the treatment variable was not imputed. Variables with greater than 40% missingness—including spirometric indices (FEV1, DLCO), pathological stage, and the six-minute-walk category—were excluded from the propensity-score model and from covariate adjustment, because imputation under such missingness is unreliable and the patients with complete spirometry constitute a non-representative subset. The percentage of missing data for each baseline covariate is reported in Supplementary Table S1; missingness exceeded 40% only for DLCO (86%), six-minute-walk category (63%), and pathological stage (48%). Imputation diagnostics—convergence of the chained-equations algorithm and comparison of observed and imputed distributions—were inspected and showed no anomalies.

Propensity scores were estimated by logistic regression on the complete baseline covariate set. Patients were matched 1:1 by nearest-neighbor matching without replacement, using a caliper of 0.20 standard deviations of the logit of the propensity score. Balance was re-assessed in the matched cohort by SMD.

The association between the intercostal block and POD1 VAS was examined with a multivariable linear regression in the matched cohort, adjusting for a parsimonious, pre-treatment covariate set (age, sex, COPD, and operation group); This combination of matching with within-sample regression adjustment was prespecified as a doubly robust estimator, which guards against bias under misspecification of either the matching or the outcome model and absorbs minor residual imbalance; it was not intended to correct for failure of matching. The unadjusted matched estimate is reported alongside the adjusted estimate to demonstrate concordance. Spirometric covariates were excluded a priori for the missingness reasons noted above. Model adequacy was confirmed by the ratio of observations to estimated parameters. Although VAS is an ordinal scale, it was modeled as continuous given its 0–10 range and approximately interval interpretation in this setting; inference was confirmed with heteroscedasticity-robust (HC3) standard errors, which did not alter the results.

Because intercostal block use was strongly patterned by operating surgeon and surgical era, we performed a sensitivity analysis adding surgeon/era to the POD1 VAS model. Given the collinearity between block and surgeon, the block’s independent contribution was assessed using an order-independent nested-model comparison testing whether block reduced residual variance beyond surgeon and era, and collinearity was quantified using the variance inflation factor.

A two-sided *p* < 0.05 defined statistical significance. Analyses were performed in R (version 4.6.0; R Foundation for Statistical Computing, Vienna, Austria) using the *mice*, *MatchIt*, *tableone*, and *cobalt* packages (with *sandwich* and *lmtest* for robust standard errors).

## 3. Results

### 3.1. Cohort and Propensity-Score Matching

**Figure 1:**
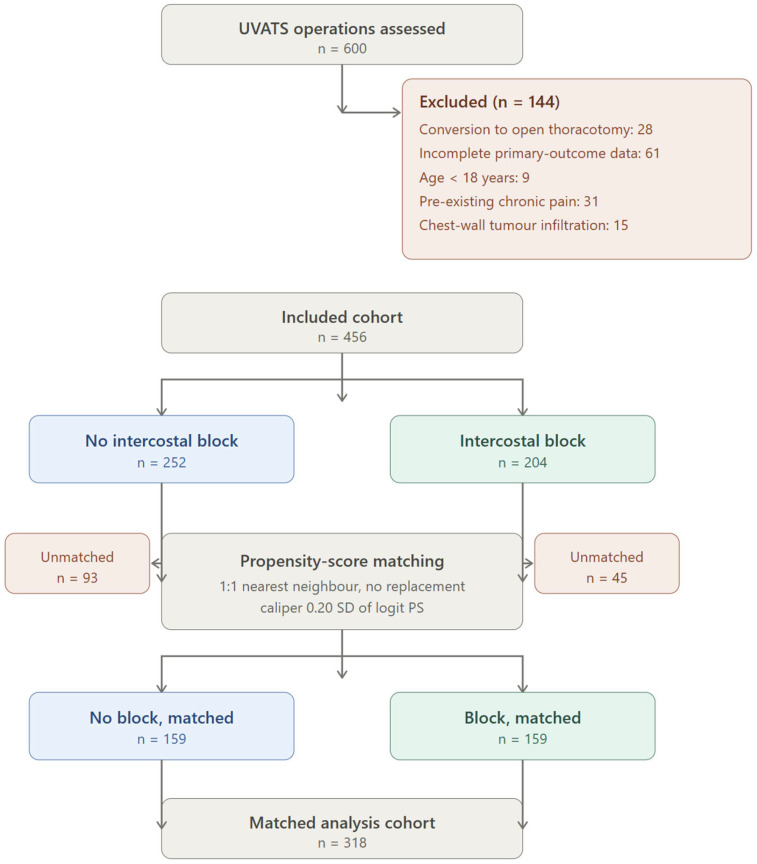
CONSORT.

Of a total of 600, 144 were excluded; 456 patients undergoing UVATS during the study period remained, of whom 252 (55.3%) received no intercostal block and 204 (44.7%) received a block. After 1:1 nearest-neighbor matching (caliper 0.20 SD of the logit propensity score), 159 patients per group were retained, yielding a matched cohort of 318 (Figure 1). Before matching, the groups were broadly comparable, with the only notable imbalance being in COPD (35.3% vs. 27.0%; SMD 0.181) (Supplementary Table S1). After matching, all clinically relevant covariates were well balanced (all SMD < 0.13), including age, sex, BMI, smoking, COPD (28.9% vs. 30.2%; SMD 0.028), diabetes, ASA class, and operation group (Table 3). Operative and intraoperative characteristics—arterial-line use, operative time, conversion to intubation, and procedure type—were also balanced after matching (Table 4), indicating that the comparison reflects the block itself rather than differences in operative management.

### 3.2. Postoperative Pain

Early pain was lower in the block group. Median POD1 VAS was 4 (IQR 3–4) with the block versus 5 (IQR 5–7) without (*p* < 0.001; SMD 1.03), and fewer block patients reported any pain on POD1–2 (71.1% vs. 81.1%; *p* = 0.049) (Table 2). By POD3 and beyond the difference narrowed: median VAS was 0 in both groups (IQR 0–3 vs. 0–4; *p* = 0.017), and the proportion with persistent pain did not differ (50.3% vs. 45.9%; *p* = 0.501). Overall pain duration was shorter with the block (median 1 [IQR 1–6.5] vs. 2 [IQR 1–9] days; *p* = 0.005).

### 3.3. Analgesic and Opioid Use

**Figure 2:**
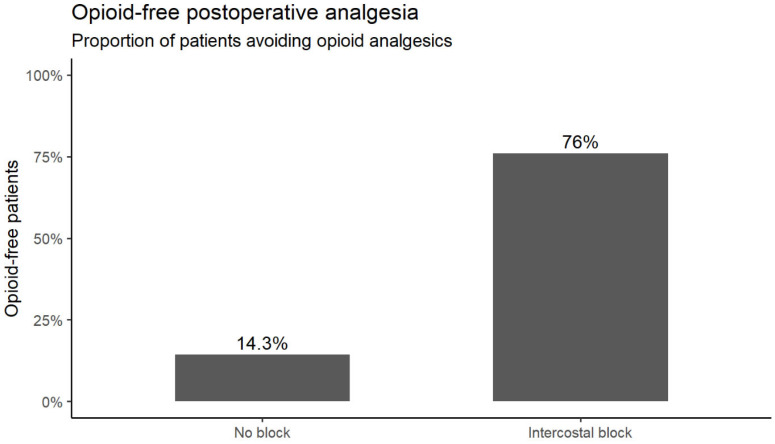
Postoperative opioid use, matched cohort. Proportion of patients managed without any postoperative ward opioid (“opioid-free”): 76.1% with the intercostal block versus 10.7% without (*p* < 0.001; standardized mean difference 1.76).

**Figure 3:**
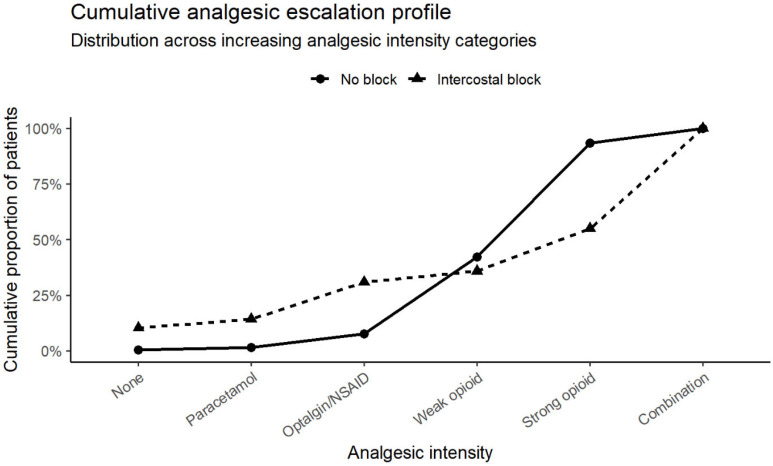
Cumulative distribution of postoperative analgesic intensity, matched cohort (*n* = 159 per group). Analgesic regimens are ranked by increasing intensity (none → paracetamol → Optalgin/NSAID → weak opioid → strong opioid → combination); curves show the cumulative proportion of patients in each category. The intercostal-block curve (dashed, triangles) lies above the no-block curve (solid, circles) across the lower-intensity categories, indicating a shift toward less intensive, predominantly non-opioid analgesia. In the block group, the “combination” category largely represents multimodal non-opioid regimens (NSAID plus paracetamol) rather than escalation to stronger agents.

Opioid exposure was markedly reduced with the block. Postoperative opioid-free management was achieved in 76.1% of block patients versus 10.7% without (*p* < 0.001; SMD 1.76) (Figure 2). The distribution of analgesic regimens differed substantially (*p* < 0.001): block patients more often received non-opioid or multimodal combinations, whereas no-block patients relied predominantly on weak and strong opioids (Table 2). The cumulative analgesic-intensity profile showed a consistent shift toward less intensive, predominantly non-opioid analgesia in the block group across all categories (Figure 3). Route of administration also differed, with less reliance on intravenous analgesia in the block group (*p* < 0.001).

### 3.4. Early Pulmonary and Other Outcomes

**Figure 4:**
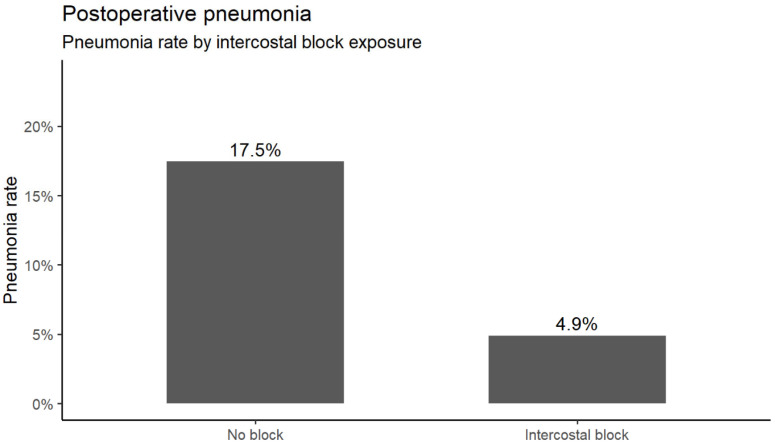
Postoperative pneumonia, matched cohort. Incidence of postoperative pneumonia: 5.7% with the intercostal block versus 20.1% without (*p* < 0.001; standardized mean difference 0.44).

Postoperative pneumonia, a prespecified secondary endpoint, was recorded less frequently in the block group (9/159 [5.7%] vs. 32/159 [20.1%]; odds ratio 0.24, 95% CI 0.11–0.52; *p* < 0.001) (Table 2, Figure 4). Pulmonary-toilet-related events did not differ (11.3% vs. 13.8%; *p* = 0.612). Thirty-day mortality (13.2% vs. 9.4%; *p* = 0.376) and one-year mortality (17.1% vs. 10.1%; *p* = 0.101) did not differ significantly (Table 5).

Thirty-day mortality (13.2% vs. 9.4%; *p* = 0.376) and one-year mortality (17.1% vs. 10.1%; *p* = 0.101) did not differ significantly (Table 5); these comparisons are descriptive, as the study was not powered for mortality and no formal time-to-event analysis was performed. In a logistic model additionally adjusting for operating surgeon and surgical era, the block–pneumonia association was not stably estimable: the model was limited by the small number of events (*n* = 41) and by collinearity between block and surgeon/era, precluding a reliable adjusted odds ratio.

### 3.5. Multivariable Analysis of POD1 Pain

**Figure 5:**
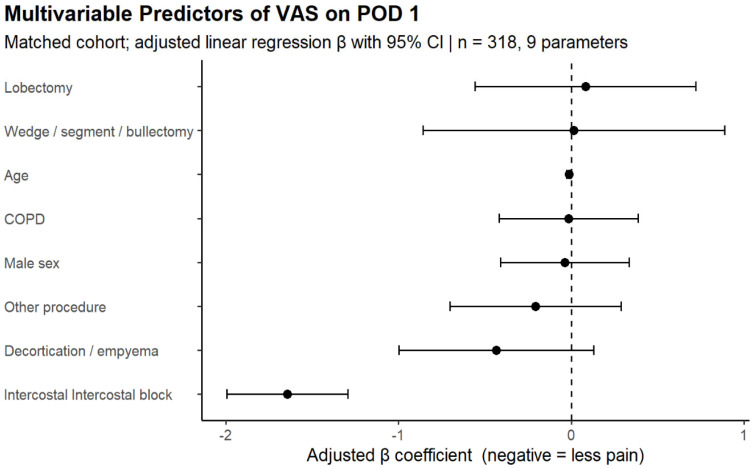
Multivariable predictors of VAS on postoperative day 1, matched cohort (*n* = 318). Points are adjusted β coefficients from a multivariable linear regression and horizontal bars the 95% confidence intervals; the dashed vertical line at 0 denotes no association, with negative values indicating lower pain. In this base model (adjusted for age, sex, COPD, and operation group but not for operating surgeon or surgical era), the intercostal block was associated with lower POD1 VAS (β = −1.64, 95% CI −2.00 to −1.29; *p* < 0.001). Older age was associated with marginally lower pain (β = −0.014 per year; *p* = 0.018); sex, COPD, and operation group were not independently associated with POD1 VAS. After additional adjustment for operating surgeon and surgical era, the block remained associated with lower POD1 VAS (β = −1.45, 95% CI −2.18 to −0.72; *p* < 0.001; see text).

In a multivariable linear regression on the matched cohort (*n* = 318; 35 observations per parameter), the block was associated with lower POD1 VAS after adjustment for age, sex, COPD, and operation group (adjusted β = −1.64, 95% CI −2.00 to −1.29; *p* < 0.001), corresponding to a reduction of approximately 1.6 points on the 0–10 scale (Figure 5). Older age was associated with marginally lower POD1 pain (β = −0.014 per year; *p* = 0.018); sex, COPD, and operation group were not independently associated with POD1 VAS. The model was significant overall (F[8, 309] = 11.8; *p* < 0.001; adjusted R^2^ = 0.21), and the block estimate was stable across alternative specifications.

Block use was strongly associated with operating surgeon and surgical era. After adding surgeon/era to the model (analytic *n* = 451 with complete data for all model covariates), the block remained associated with lower POD1 VAS (β = −1.45, 95% CI −2.18 to −0.72; *p* < 0.001), and an order-independent nested-model comparison confirmed that the block explained variance in POD1 pain beyond surgeon and era (incremental F = 15.2; *p* = 0.0001). The surgeon term was not independently associated with POD1 VAS after adjustment (β = −0.58; *p* = 0.122). Block and surgeon were moderately collinear (variance inflation factor ≈ 8.4), which widened the confidence interval but did not abolish the association.

## 4. Discussion

In this propensity-score-matched, single-center cohort of UVATS patients, a simple surgeon-administered intercostal nerve block was associated with clinically meaningful reductions in early postoperative pain. The early POD1 VAS reduction (≈1.5 points after adjustment for surgeon and era) closely reproduces the pooled estimate of the largest meta-analysis in this field, in which ICNB lowered dynamic pain by 1.66 points at 0–6 h and 1.27 points at 7–24 h versus systemic analgesia [4]. That a single-center result aligns with the pooled multi-center data suggests external concordance with the early analgesic association; however, the single-center, retrospective design and the operator/era confounding detailed below limit causal and external-validity claims. The opioid-sparing magnitude we observed—76% versus 11% opioid-free—is consistent with the opioid-reduction and faster-recovery signals reported in recent randomized trials of surgeon-placed ICNB [5] and with the reported noninferiority of ICNB to TEA for pain and length of stay [6], although, as discussed below, this endpoint was confounded with surgical era in our cohort. The magnitude of the opioid-sparing effect warrants cautious interpretation. The between-group difference in opioid-free management (65 percentage points) was substantially larger than the corresponding difference in POD1 VAS (≈1 point), and an analgesic mechanism would be expected to move the two endpoints concordantly. This disproportion, together with the low opioid-free proportion among unblocked patients despite only moderate recorded pain, indicates that opioid prescriptions were influenced by factors beyond the documented pain score. In a non-randomized design with an unblinded treating team and discretionary rescue analgesia, knowledge of block status can shape prescribing independently of pain intensity. We therefore interpret the opioid-free endpoint as reflecting both a genuine analgesic effect and a treatment-associated prescribing pattern, and we regard the POD1 VAS reduction—which is less susceptible to such influence—as the more robust estimate of the analgesic effect of the block.

The role of ICNB relative to other regional techniques nonetheless remains contested, and our findings should not be read as establishing it as the optimal block. The PROSPECT guideline does not recommend ICNB as a first-line technique, citing insufficient procedure-specific evidence and favoring paravertebral or erector spinae plane blocks [2]. Randomized and systematic data—including a recent review from our region—rank PVB superior to ICNB for 24 h pain and opioid consumption [7,8], and serratus anterior plane block has shown longer-lasting relief at 24–48 h [3]. Our results are best interpreted within this context: a simple, surgeon-administered, single-shot block delivers a clinically meaningful early analgesic association within a real-world UVATS pathway, without the technical demands, ultrasound dependence, or neuraxial risks of its alternatives. For many centers, that pragmatic profile—not theoretical superiority—is the relevant consideration.

The temporal profile of our results carries a specific mechanistic and clinical implication. The robust POD1 effect that attenuated by POD3 mirrors the established pharmacology of single-shot ICNB, whose analgesic window is limited to approximately 6–8 h and dissipates by 24–48 h, with documented vulnerability to rebound pain [4,10]. The numerically higher prevalence of POD3+ pain in the block group is consistent with this rebound phenomenon rather than with any deleterious effect of the block. This temporal limitation also bears on patients who develop prolonged postoperative pain. The available evidence indicates that single-shot ICNB does not reduce the incidence of chronic post-thoracotomy pain [3,11,12], and that preemptive local blockade—unlike thoracic epidural analgesia—has not been shown to prevent the chronic pain syndrome. The demonstrable benefit of ICNB instead lies in the acute phase, where preserved respiratory mechanics and reduced splinting are associated with fewer pulmonary complications (reported odds ratio approximately 0.45 for postoperative pulmonary complications versus systemic analgesia) [4]. We therefore interpret the lower pneumonia rate in our block group as an acute, mechanically mediated association rather than a definitively causal reduction. Its magnitude (odds ratio ≈ 0.24) is larger than the pooled estimate for pulmonary complications (≈0.45) [4], which may reflect the comorbidity-laden, heterogeneous case-mix of a consecutive uniportal cohort—in which decortication and diagnostic indications are well represented—rather than a block effect alone. Reassuringly, the confounders most likely to drive such a difference (age, COPD, ASA class, and operative time) were closely balanced after matching (Table 2 and Table 3), so the association is not attributable to these factors; however, baseline pulmonary reserve was incompletely captured and remains a potential residual confounder. Consistent with this, the pneumonia association could not be reliably estimated once operating surgeon and surgical era were taken into account, owing to the small number of events and the collinearity between block and surgeon. We therefore regard the pneumonia finding as exploratory and hypothesis-generating, and do not interpret it as a demonstrated causal effect of the block.

In patients who went on to experience prolonged pain, the block is unlikely to have altered the chronic pain trajectory and more plausibly mitigated early respiratory morbidity.

These observations are clarified by distinguishing the two components of post-thoracoscopic pain. Somatic chest-wall pain arises from the utility incision, intercostal nerve trauma, and rib and soft-tissue retraction; in a single-incision approach, this load is not diminished and may be concentrated at the instrumented level, where the convergence of camera and instruments imposes sustained compression and leverage on the intercostal nerve. Visceral pain, by contrast, arises from lung manipulation and pleural dissection and is largely independent of the number of ports. A surgeon-placed intercostal block acts on the somatic component at the targeted levels and would not be expected to modify visceral nociception. This framework is in accordance with our temporal findings: the block was associated with lower early pain, when somatic chest-wall nociception predominates, and the effect attenuated by POD3 as this component subsides and the single-shot pharmacological window closes, leaving residual later pain plausibly of visceral or non-somatic origin. It also explains why a level-targeted block retains analgesic value in UVATS despite the reduced number of incisions.

The chief strength of this study is its design. By drawing on a single center with a standardized UVATS technique, a uniform anesthetic agent and block protocol, and a single perioperative pathway, we reduce the inter-institutional heterogeneity in surgical approaches, anesthetic mixture, and block timing that limits pooled comparative analyses [5,13,14,15,16]. Propensity-score matching achieved balance not only on baseline comorbidity but also on operative and airway-management variables. However, this design does not overcome the central limitation described below: because block use was adopted contemporaneously with a change in operating surgeon and surgical era, the matched comparison cannot be read as reflecting the block alone, and the analgesic association—though robust to adjustment for surgeon and era—remains observational. This study has several limitations. First, it is retrospective and nonrandomized; although propensity-score matching balanced measured covariates, residual confounding by indication cannot be excluded. This is most relevant to the nonsignificant trend toward higher one-year mortality in the block group, which was directionally consistent before and after matching and most plausibly reflects preferential use of the regional approach in frailer or more palliative patients rather than any harm attributable to the block; the study was underpowered for this outcome, and no survival inference should be drawn. With 36 and 43 deaths at 30 days and one year, respectively, the study was underpowered for these endpoints; exact dates of death were not uniformly available, so formal time-to-event analysis was not undertaken and mortality is reported as crude proportions at fixed time points. Second, the opioid-use endpoints are susceptible to co-intervention (ascertainment) bias: prescribing decisions were made by an unblinded treating team under a discretionary multimodal protocol, so knowledge of block status may have influenced opioid administration independently of pain intensity, and the opioid-use variables were in any case defined only on the postoperative ward window. This bias acts after treatment assignment and is not removed by propensity-score matching; it most directly qualifies the opioid-free endpoint, which should be read as a combined pharmacological and practice-pattern effect rather than an unbiased pharmacological estimate. opioid exposure was, moreover, available only categorically (opioid-free status and specific agent) rather than as morphine milligram equivalents, precluding a quantitative dose comparison. Third, the analgesic effect of a single-shot block is, by pharmacology, confined to the early postoperative window; our data cannot speak to durable or chronic pain outcomes. Fourth, spirometric indices (FEV1, DLCO), pathological stage, and functional-capacity data were missing in a substantial proportion of patients (DLCO 86%, six-minute-walk, 63% non-estimable, stage 48%) and were therefore excluded from the propensity model and adjustment; unmeasured baseline pulmonary function and disease stage, which plausibly influence both block allocation and pulmonary and survival outcomes, are potential residual confounder that cannot be excluded; this most directly qualifies the pneumonia and mortality association, which should accordingly be regarded as exploratory and hypothesis-generating. In addition, postoperative pneumonia was ascertained retrospectively by an unblinded clinical team rather than by independent blinded adjudication, so ascertainment bias for this endpoint cannot be excluded. Fifth, length-of-stay and readmission data were not of sufficient quality to report and were omitted, limiting assessment of recovery beyond pain and opioid endpoints. Sixth, most consequentially, intercostal block use was strongly patterned by operating surgeon and surgical era, raising the possibility of operator-related and historical confounding. In a sensitivity analysis adding surgeon/era, the early analgesic association persisted and was confirmed by an order-independent nested-model test, although block and surgeon were moderately collinear (variance inflation factor ≈ 8.4), limiting the precision of the adjusted estimate. Because block allocation was non-randomized and correlated with surgeon preference, residual confounding cannot be fully excluded; this caveat applies most strongly to the opioid-free and pneumonia endpoints, which were more closely aligned with secular trend and for which an independent block effect could not be reliably established. Finally, the single-center design, while controlling heterogeneity, limits generalizability to centers using different agents, block timing, or surgical approaches.

## 5. Conclusions

In uniportal VATS, a simple surgeon-administered intercostal nerve block was associated with a clinically meaningful reduction in early postoperative pain that persisted after adjustment for operating surgeon and surgical era and was confirmed by an order-independent test, despite moderate collinearity between block use and surgeon. The magnitude of this early analgesic association is concordant with pooled meta-analytic estimates and with the known pharmacology of single-shot blockade, which confines the effect to the acute postoperative window. The accompanying reductions in opioid use and postoperative pneumonia were more closely aligned with surgeon and secular trend and did not prove to be robustly independent of these factors; they should be regarded as hypothesis-generating. Within these limits, the intercostal block is a pragmatic, low-risk option for early analgesia in UVATS, but its opioid-sparing and pulmonary benefits require confirmation in prospective, protocol-randomized studies with concurrent operators.

## Figures and Tables

**Table 1:** The study question is summarized in PECO format.

Element	Definition
**Population (P)**	Adults undergoing uniportal VATS for any indication at a single center (2017–2025)
**Exposure (E)**	Standardized surgeon-administered intercostal nerve block at three levels, placed under thoracoscopic vision before closure
**Comparator (C)**	Same UVATS pathway and multimodal analgesia without intercostal block
**Outcomes (O)**	Primary: POD1 pain (VAS) and postoperative opioid-free management. Secondary: later pain, analgesic regimen, postoperative pneumonia, mortality

**Table 2:** Postoperative pain, analgesic use, and early pulmonary outcomes, matched cohort. Values are *n* (%) or median [IQR]. *p*-values: Mann–Whitney U (continuous), chi-square/Fisher exact (categorical); multilevel rows tested as omnibus. “Opioid-free” denotes absence of postoperative ward opioid administration.

Variable	No Block (*n* = 159)	Block (*n* = 159)	*p*	SMD
Pain				
Pain present POD1–2	129 (81.1)	113 (71.1)	0.049	0.238
VAS POD1–2	5 [5–7]	4 [3–4]	<0.001	1.031
Pain present POD3+	73 (45.9)	80 (50.3)	0.501	0.088
VAS POD3+	0 [0–4]	0 [0–3]	0.017	0.301
Pain duration, d	2 [1–9]	1 [1–6.5]	0.005	0.250
Analgesic class			<0.001	1.811
—None	0 (0.0)	14 (8.8)		
—Paracetamol	2 (1.3)	8 (5.0)		
—Optalgin/NSAID	7 (4.4)	26 (16.4)		
—Weak opioid	58 (36.5)	9 (5.7)		
—Strong opioid	84 (52.8)	29 (18.2)		
—Combination (multimodal)	8 (5.0)	73 (45.9)		
Specific opioid			<0.001	2.281
—None	17 (10.7)	121 (76.1)		
—Tramadol	58 (36.5)	7 (4.4)		
—Zaldiar	0 (0.0)	2 (1.3)		
—Percocet	28 (17.6)	1 (0.6)		
—Oxycodone	36 (22.6)	1 (0.6)		
—Morphine	1 (0.6)	2 (1.3)		
—Fentanyl †	19 (11.9)	25 (15.7)		
Opioid-free	17 (10.7)	121 (76.1)	<0.001	1.756
Route (none/PO/PO + IV), %	0/24.5/75.5	8.8/27.0/64.2	<0.001	0.455
Pulmonary				
Postoperative pneumonia	32 (20.1)	9 (5.7)	<0.001	0.442
Pulmonary toilet-related	22 (13.8)	18 (11.3)	0.612	0.076

† Fentanyl denotes postoperative ward administration; intraoperative anesthetic fentanyl was not counted. Absolute risk difference and number needed to treat (NNT): opioid-free management, ARR 65.4%, NNT 2; postoperative pneumonia, ARR 14.4%, NNT 7. As these endpoints were not robust to adjustment for surgeon/era, the NNT values are descriptive and should not be interpreted causally.

**Table 3:** Baseline characteristics, matched cohort. Values are *n* (%) or median [IQR]. Balance is assessed by standardized mean difference (SMD); SMD < 0.20 indicates adequate balance.

Characteristic	No Block (*n* = 159)	Block (*n* = 159)	SMD
Age, y	69 [59–80.5]	70 [59–79.5]	0.008
Male sex	91 (57.2)	90 (56.6)	0.013
BMI	25.0 [21.8–29.0]	24.8 [21.8–28.6]	0.015
Smoking	87 (54.7)	83 (52.2)	0.050
Hypertension	96 (62.7)	102 (66.2)	0.073
Diabetes mellitus	57 (35.8)	54 (34.0)	0.040
Ischemic heart disease	48 (30.2)	45 (28.3)	0.041
COPD	46 (28.9)	48 (30.2)	0.028
Chronic renal failure	19 (11.9)	19 (11.9)	<0.001
ASA (I/II/III/IV), %	1.3/22.6/57.2/18.9	0.6/23.3/57.2/18.9	0.066
Malignancy	86 (54.1)	85 (53.5)	0.013
Mallampati (I/II/III/IV), %	23.3/69.3/6.7/0.7	27.3/64.3/7.0/1.4	0.124

**Table 4:** Operative and intraoperative characteristics, matched cohort. Values are *n* (%) or median [IQR].

Variable	No Block (*n* = 159)	Block (*n* = 159)	SMD
Arterial line	24 (15.1)	20 (12.6)	0.073
Operative time, min	43 [30–68]	42 [28–65]	0.065
Conversion to intubation	82 (51.6)	73 (45.9)	0.113
Need for intubation	74 (46.5)	81 (50.9)	0.088
Operation group (biopsy/decort/lobe/wedge/other), %	20.8/20.8/12.6/6.9/39.0	26.4/20.1/10.7/5.7/37.1	0.143

**Table 5:** Mortality, matched cohort.

Outcome	No Block (*n* = 159)	Block (*n* = 159)	*p*	SMD
30-day mortality	15 (9.4)	21 (13.2)	0.376	0.119
1-year mortality	16 (10.1)	27 (17.1)	0.101	0.204

## Data Availability

The data presented in this study are available on request from the corresponding author.

## Supplementary Materials

The following supporting information can be downloaded at: https://www.mdpi.com/article/10.3390/jcm15134910/s1. Supplementary Table S1. Baseline characteristics before matching. Supplementary Table S2. Operative and intraoperative characteristics before matching. Supplementary Table S3. Postoperative pain, analgesic use, and early pulmonary outcomes before matching. Supplementary Table S4. Mortality before matching.
