# Supplementary material for: Intercostal Nerve Block in Uniportal Video-Assisted Thoracoscopic Surgery: A Propensity-Score Matched Single-Center Study of Early Postoperative Pain and Opioid Use
Source: J Clin Med. 2026 Jun 24;15(13):4910. doi: 10.3390/jcm15134910 (PMC13361061; doi:10.3390/jcm15134910)
Supplement: Supplementary file 1 [file jcm-15-04910-s001.zip › jcm-4399920-supplementary.pdf]

Supplementary Table S1. Baseline characteristics before matching. *Values are n (%) or median [IQR]. P-values: Mann–Whitney U (continuous), chi-square/Fisher exact (categorical). High-missingness variables (DLCO, stage, six-minute-walk) are shown for completeness but were excluded from the propensity-score model; their SMDs reflect missingness artefact rather than true imbalance.*

Characteristic	No block (n=252)	Block (n=204)	p	SMD
Age, y	67 [58–77]	71 [59–79]	0.153	0.122
Male sex	134 (53.2)	116 (56.9)	0.489	0.074
BMI	25.0 [22.2–28.9]	25.6 [22.1–29.4]	0.395	0.085
Smoking	139 (55.2)	103 (50.5)	0.369	0.094
Hypertension	140 (58.3)	123 (63.7)	0.296	0.111
Diabetes mellitus	88 (34.9)	61 (29.9)	0.300	0.107
Ischemic heart disease	68 (27.0)	50 (24.5)	0.622	0.057
COPD	89 (35.3)	55 (27.0)	0.071	0.181
Chronic renal failure	26 (10.3)	28 (13.7)	0.330	0.105
Liver disease	12 (4.9)	10 (5.2)	1.000	0.017
Rheumatic disease	2 (0.8)	5 (2.6)	0.266	0.140
Sarcoidosis	11 (4.4)	6 (2.9)	0.583	0.076
Alcohol abuse	10 (4.2)	7 (3.6)	0.971	0.028
Tuberculosis (none/active/S-P), %	96.7/2.9/0.4	97.4/2.1/0.5	0.849	0.056
Chronic steroids	18 (7.3)	14 (7.3)	1.000	0.002
ASA (I/II/III/IV), %	1.2/27.3/53.8/17.7	0.5/21.5/58.6/19.4	0.447	0.159
FEV1, %	69 [44.5–88]	68.5 [48.3–88.8]	0.727	0.075
DLCO, %‡	86 [72–95]	87 [70–97]	0.483	0.022
6-min walk (<4/4–7/>7/NE), %‡	15.4/19.8/8.1/56.7	13.1/12.0/3.7/71.2	0.010	0.334
Malignancy	140 (56.7)	110 (57.6)	0.925	0.018

Characteristic	No block (n=252)	Block (n=204)	p	SMD
Stage‡	—	—	0.316	0.462
Mallampati (I/II/III/IV), %	25.7/65.8/8.0/0.4	25.7/65.4/7.8/1.1	0.875	0.080
Hemodynamically stable	246 (97.6)	202 (99.0)	0.439	0.109

‡High missingness (DLCO 86%, six-minute-walk 63% not-estimable, stage 48%); descriptive only, excluded from the propensity model.

Supplementary Table S2. Operative and intraoperative characteristics before matching.

Variable	No block (n=252)	Block (n=204)	p	SMD
Arterial line	83 (32.9)	20 (9.8)	<0.001	0.588
Operative time, min	48 [31.8–105]	42 [28.5–61.5]	0.006	0.337
Conversion to intubation	166 (65.9)	82 (40.2)	<0.001	0.532
Need for intubation	105 (41.7)	104 (51.0)	0.059	0.188
Operation group (biopsy/decort/lobe/wedge/other), %	19.8/18.7/14.7/6.7/40.1	27.0/18.6/9.3/7.4/37.7	0.254	0.220

Note: the large pre-matching imbalances in arterial-line use, operative time, and conversion to intubation reflect that the block was preferentially used within a less invasive anesthetic/airway approach; all three were balanced after matching (Table 2).

Supplementary Table S3. Postoperative pain, analgesic use, and early pulmonary outcomes before matching.

Variable	No block (n=252)	Block (n=204)	p	SMD
Pain present POD1–2	184 (73.0)	142 (69.6)	0.486	0.075
VAS POD1–2	5 [5–7]	4 [3–4]	<0.001	0.949
Pain present POD3+	105 (41.7)	103 (50.5)	0.074	0.178

**Variable**	**No block (n=252)**	**Block (n=204)**	**p**	**SMD**
VAS POD3+	0 [0–4]	0 [0–3]	0.072	0.210
Pain duration, d	2 [1–7]	1 [1–5]	<0.001	0.274
**Analgesic class**			<0.001	1.649
— None	1 (0.4)	21 (10.3)		
— Paracetamol	3 (1.2)	8 (3.9)		
— Optalgin/NSAID	15 (6.0)	34 (16.7)		
— Weak opioid	87 (34.5)	10 (4.9)		
— Strong opioid	129 (51.2)	39 (19.1)		
— Combination	17 (6.7)	92 (45.1)		
**Specific opioid**			<0.001	1.977
— None	36 (14.3)	155 (76.0)		
— Tramadol	87 (34.5)	8 (3.9)		
— Zaldiar	0 (0.0)	2 (1.0)		
— Percocet	43 (17.1)	1 (0.5)		
— Oxycodone	43 (17.1)	1 (0.5)		
— Morphine	2 (0.8)	2 (1.0)		
— Fentanyl†	41 (16.3)	35 (17.2)		
**Opioid-free**	36 (14.3)	155 (76.0)	<0.001	1.580
**Route** (none/PO/PO+IV), %	0.4/19.0/80.6	10.3/27.5/62.3	<0.001	0.528
Postoperative pneumonia	44 (17.5)	10 (4.9)	<0.001	0.407
Pulmonary toilet–related	33 (13.1)	23 (11.3)	0.656	0.056

Supplementary Table S4. Mortality before matching.

Outcome	No block (n=252)	Block (n=204)	p	SMD
30-day mortality	25 (9.9)	25 (12.4)	0.497	0.078
1-year mortality	25 (10.0)	34 (16.9)	0.041	0.205

Note: the pre-matching one-year mortality difference (p=0.041) attenuated to non-significance after matching (Table 4); its persistence in direction is discussed as probable residual confounding by indication.
